# Acute amnestic syndrome in fornix lesions: a systematic review of reported cases with a focus on differential diagnosis

**DOI:** 10.3389/fneur.2024.1338291

**Published:** 2024-01-25

**Authors:** F. Mazzacane, F. Ferrari, A. Malvaso, Y. Mottese, M. Gastaldi, A. Costa, A. Pichiecchio, A. Cavallini

**Affiliations:** ^1^Department of Brain and Behavioral Sciences, University of Pavia, Pavia, Italy; ^2^Department of Emergency Neurology and Stroke Unit, IRCCS Fondazione Mondino, Pavia, Italy; ^3^Unit of Behavioral Neurology, Mondino Foundation, Pavia, Italy; ^4^Neuroimmunology Unit, IRCCS Mondino Foundation, Pavia, Italy; ^5^Department of Neuroradiology, IRCCS Mondino Foundation, Pavia, Italy

**Keywords:** amnesia, fornix, stroke, review, limbic system

## Abstract

**Introduction:**

Acute amnestic syndrome is an uncommon clinical presentation of neurological disease. Differential diagnosis encompasses several syndromes including Wernicke-Korsakoff and transient global amnesia (TGA). Structural lesions of the fornix account for a minority of cases of acute amnestic syndromes. Etiology varies from iatrogenic injury to ischemic, inflammatory, or neoplastic lesions. A prompt diagnosis of the underlying pathology is essential but challenging. The aim of this review is to systematically review the existing literature regarding cases of acute amnestic syndrome associated with non-iatrogenic lesions of the fornix.

**Methods:**

We performed a systematic literature search on PubMed, Scopus, and Web of Science up to September 2023 to identify case reports and case series of patients with amnestic syndrome due to fornix lesions. The systematic review was conducted according to PRISMA guidelines. The research was limited to articles written in English. Cases of fornix damage directly ascribable to a surgical procedure were excluded.

**Results:**

A total of 52 publications reporting 55 cases were included in the review. Focusing on acute/subacute onset, vascular etiology was highly prevalent, being responsible for 78% of cases, 40/55 (74%) of which were due to acute ischemic stroke. The amnestic syndrome was characterized by anterograde amnesia in all patients, associated with retrograde amnesia in 27% of cases. Amnesia was an isolated presentation in most cases. Up to two thirds of patients had persistent memory deficits of any severity at follow-up.

**Discussion:**

Acute amnestic syndrome can be rarely caused by fornix lesions. In most cases of acute/subacute presentation, the etiology is ischemic stroke, mainly caused by strokes involving the subcallosal artery territory. The differential diagnosis is challenging and a distinction from common mimics is often difficult on a clinical basis. A high index of suspicion should be maintained to avoid misdiagnosis and provide adequate acute treatment to patients with time-dependent disease, also employing advanced neuroimaging. More research is needed to better understand the outcome and identify prognostic factors in patients with amnestic syndrome due to fornix lesions.

## Introduction

Acute amnestic syndrome is an uncommon acute clinical presentation of neurological disease (1). Differential diagnosis encompasses many disorders such as transient global amnesia (TGA) and Wernicke-Korsakoff syndrome; moreover, the involvement of different anatomical structures can be responsible of this neurological syndrome (1).

**Figure 1:**
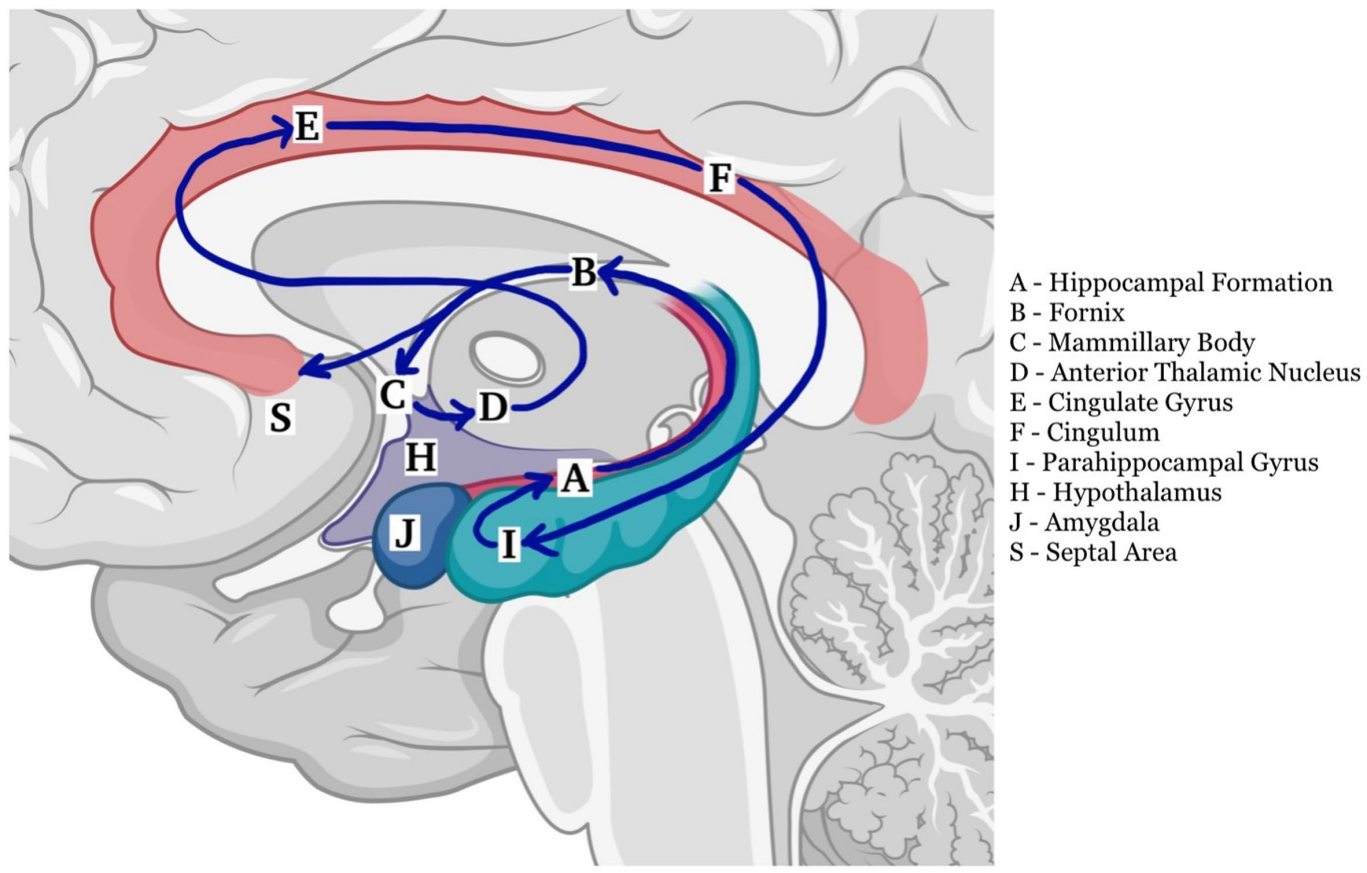
The Papez circuit plays a critical role in the processing and transfer of information for long storage (2). It begins in the hippocampus (A) and continues into the fornix (B) to reach the mamillary body (C), and then through the mammillothalamic tract continues to the anterior nucleus of the thalamus (D). From there it connects to the cingulate gyrus (E) by means of anterior thalamic radiations. The cingulum (F) courses around the corpus callosum to reach the entorhinal cortex (I), which projects to the hippocampus (A), closing the circuit (3) (created with BioRender.com).

**Figure 2:**
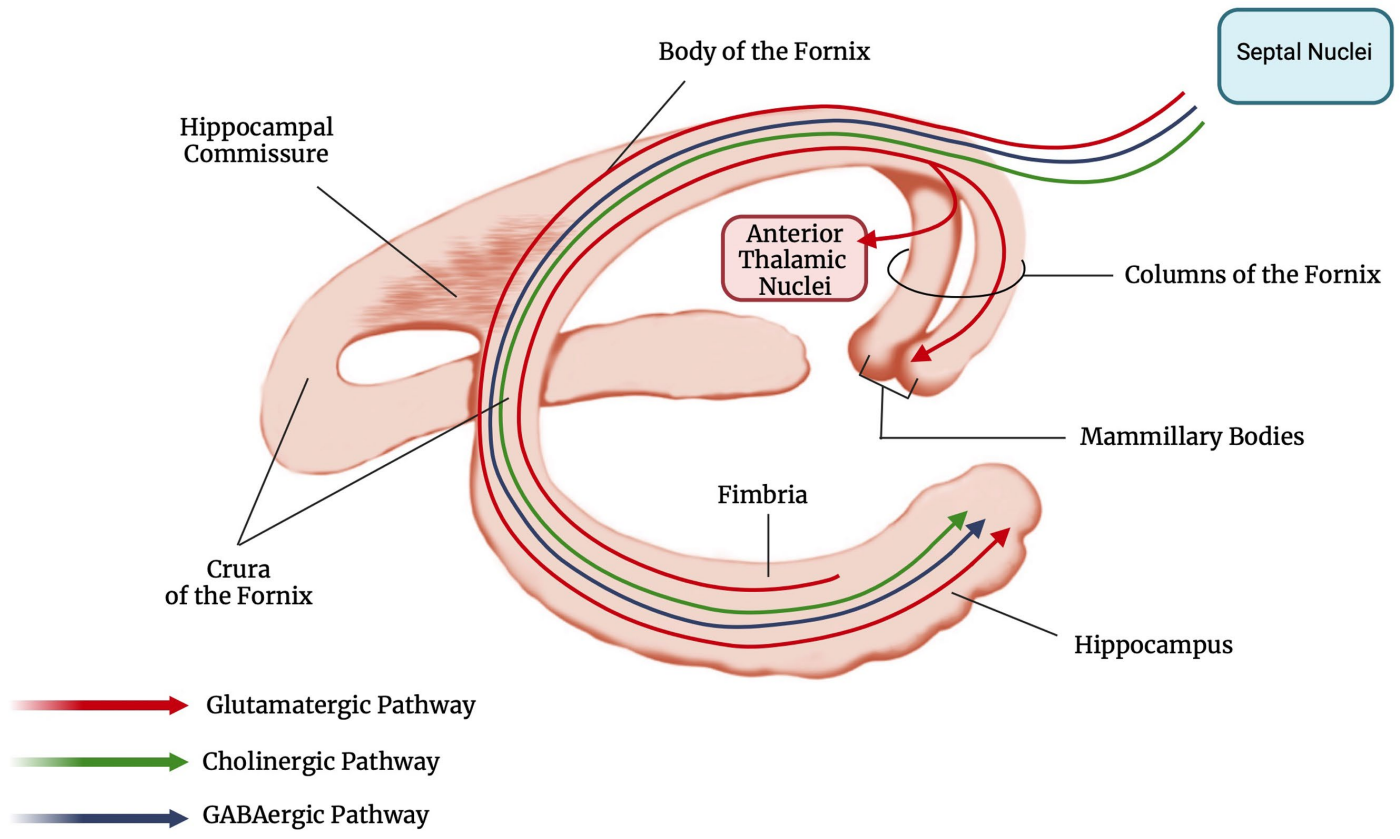
Anatomy and main neural connections of the fornix (created with BioRender.com).

The fornix is both a commissure, a projection, and an association tract, mainly composed of fibers that represent the major hippocampal output. It has a key role within the Papez circuit (Figure 1), which is central in memory storage and is a part of the cholinergic, GABAergic and glutamatergic neurotransmission systems (4). The neural and neurochemical connections of the fornix with the nearby structures are reported in Figure 2. The fornices, as a part of this extended hippocampal-diencephalic system, are thought to contribute to the efficient encoding and normal recall of new episodic information (5). Specifically, considering amnesia as a symptom, the hippocampal formation, amygdala, paralimbic cortices, medial and anterior nuclei of the thalamus, mammillary bodies, basal forebrain and ventral striatum are all critical structures. Bilateral lesions to any of these components can cause significant episodic memory deficits, predominantly anterograde amnesia (6, 7); the severity of the memory deficit is related to the volume of damage of the affected structure (8).

Fornix vascularization (Table 1) is mainly provided by the subcallosal artery (ScA), which vascularizes also the genu of the corpus callosum, the septal nuclei and anterior cingulum in some subjects (9). The medial or lateral posterior choroidal arteries arising from the posterior circulation can also be involved (10).

**Table 1:** Fornix vascularization.

Fornix portion	Artery	Arterial origin	Other supplied vascular territories
Columns of the fornix	Sub-callosal artery	ACoA	Genu of corpus callosum, septal nuclei, anterior cingulum
		A1 of ACA	
		A2 of ACA	
	Median artery of corpus callosum	ACoA	Genu of corpus callosum, septal nuclei, anterior cingulum, body of the fornix, splenium of the corpus callosum
	Medial and lateral posterior choroidal arteries	P2 of PCA	Medial: tegmentum, midbrain, posterior thalamus, pineal gland (cisternal segment), ipsilateral choroid plexus of lateral ventricle (plexal segment). Lateral: choroid plexus of the lateral ventricle, posterior part of thalamus and of caudate nucleus, occasionally the hippocampus and mesial temporal lobe. Medial and lateral arteries anastomize at the level of foramen of Monro.
		PCA branches (e.g., parieto-occiptal, calcarine, splenial artery)	
	Hypothalamic small branches	PCoA	Posterior part of hypothalamus, mammillary bodies
Crus	Lateral posterior choroidal arteries	P2 of PCA	See above
Body	Medial and lateral posterior choroidal arteries	P2 of PCA	See above
Fimbria	Medial and lateral posterior choroidal arteries	P2 of PCA	See above

The consequences of forniceal injury in humans can be seen from post-operative cases involving forniceal transection or injury. The typical syndrome includes anterograde amnesia (11–26), that can be associated with a temporally graded retrograde amnesia (particularly in cases of fornix bilateral columns damage) (27). A lateralized fornix function has also been suggested by clinical reports, with the right fornix involved in visuospatial memory and the left fornix in verbal memory (20).

Overall, structural lesions in the fornix account for a minority of cases of anterograde amnesia, and several etiologies have been reported, including ischemic, inflammatory, neoplastic, and iatrogenic lesions (21, 28–30). Due to its rarity, acute amnestic syndrome due to fornix lesions has been mainly described in case reports and short case series.

The aim of this study is to systematically review the existing literature regarding cases of acute amnestic syndrome associated with fornix lesions, with a focus on clinical features, etiology, and differential diagnosis, to provide useful information to improve the diagnosis and treatment of these patients on clinical grounds.

## Methods

This review is reported according to the PRISMA (Preferred Reporting Items for Systematic Reviews and Meta-Analyses) guidelines (31).

### Data sources and searches

We performed a systematic literature search on PubMed, Scopus, and Web of Science up to September 2023 to identify case reports/case series of patients with acute amnestic syndrome due to lesions of the fornix. The research was limited to articles written in English. Search terms are provided in Supplementary Table S1.

### Study selection

The screening of eligible publications was carried out independently by two raters. First, the titles and abstracts of all citations were reviewed. Next, the full text of potentially relevant citations was reviewed. Discrepancies were resolved by consensus.

Studies were included if they met all the following eligibility criteria: (1) acute/subacute amnestic syndrome (< 1 month); (2) amnesia was a prominent feature of the clinical picture at presentation; (3) fornix lesion confirmed by neuroimaging.

Studies were excluded if they met one or more of the following criteria: (1) fornix damage that could be directly attributed to a surgical or endovascular procedure; (2) presence of extensive damage to other limbic structures that could explain the memory deficit.

Reference lists of eligible studies were also reviewed To identify additional relevant reports. All studies were independently analyzed By two reviewers (F.M. and F.F.) and discrepancies were resolved By discussion and consensus.

### Data extraction and quality assessment

Data was extracted by one reviewer and crosschecked by another. We extracted the following data: the first author’s last name, year of publication, patients’ demographic and clinical data, amnesic syndrome characteristics, associated neurologic deficits, neuroimaging data, etiology, and outcome; for patients with ischemic stroke, vascular risk factors and stroke etiology according to Trial of Org 10,172 in Acute Stroke Treatment classification (TOAST) classification (32) were recorded.

Quality of studies included in the systematic review was independently assessed by two authors (F.M. and F.F.) using the NIH Quality Assessment Tool for Case Series Studies (33).

### Data synthesis and analysis

Data were summarized using descriptive statistics. Categorical variables were expressed as count (percentage). Continuous variables were summarized as mean (standard deviation, SD) or median (interquartile range, IQR) in case of normal and non-normal distribution, respectively.

## Results

**Figure 3:**
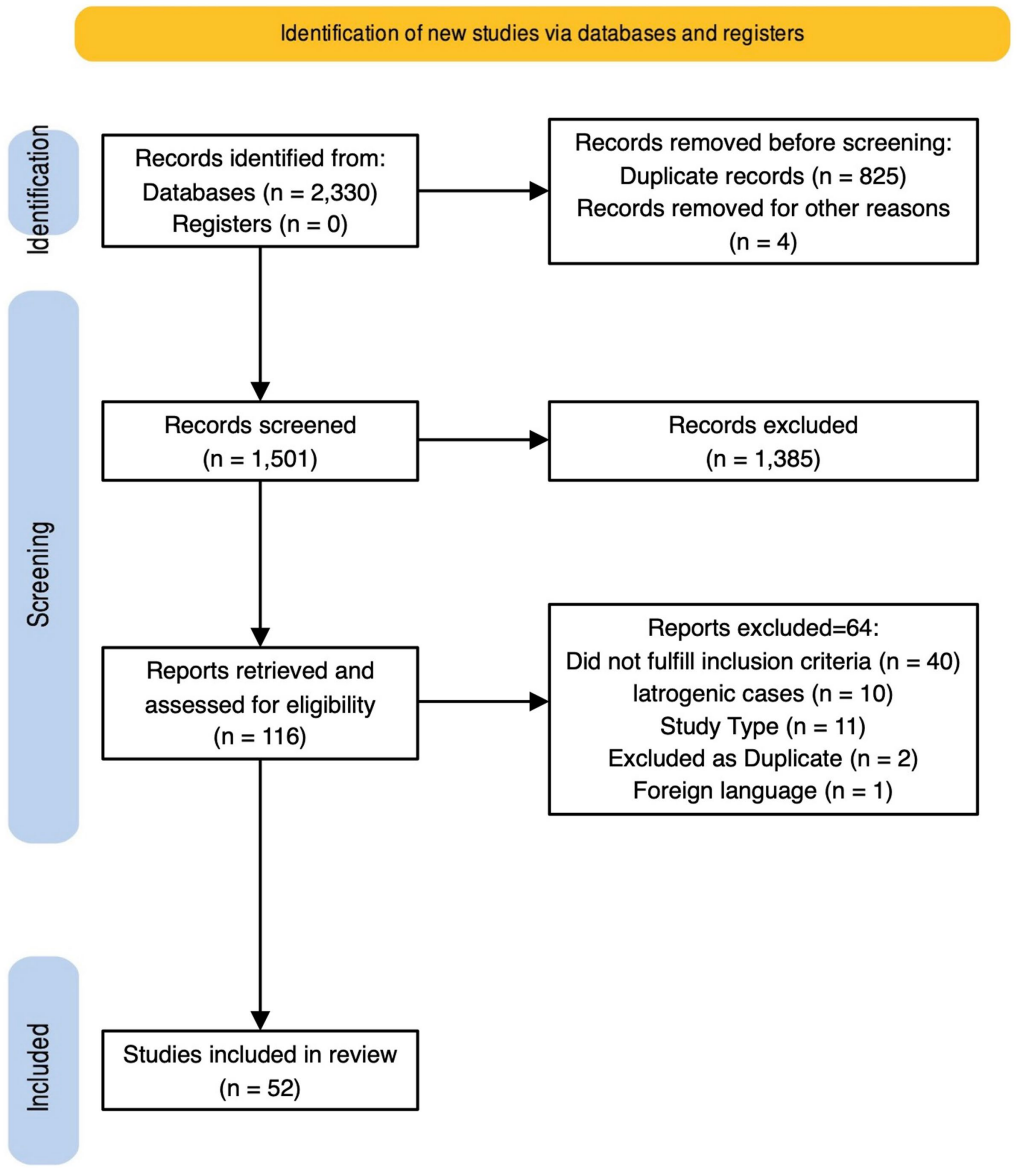
PRISMA flowchart of this systematic review.

A total of 2,330 records were initially retrieved. After duplicates removal, 1,501 records were assessed for eligibility through abstract screening. 116 were included for full text review and 52 of them, reporting 55 cases, were finally included in the review. Study selection process is described in detail in Figure 3 (31, 34), and quality assessment in Supplementary Table S2. Details on included cases are provided in Supplementary Table S3.

A total of 55 cases were included in the analysis. Twenty-seven patients (49%) were female with a mean (±SD) age of 58 (±14). As expected, focusing on acute/subacute onset, vascular etiology was highly prevalent, being responsible of 43/55 (78%) of cases, 40 (74%) of which were due to acute ischemic stroke (29, 35–71) and 3 (5%) to hemorrhagic stroke (72–75).

Neoplastic etiology was the second most common, being identified in 15% of patients. The associated amnestic syndrome was caused either by fornix infiltration [B cell lymphoma (28, 76), pineal germinoma (77), spongioblastoma (78)] or following an acute intratumoral hemorrhage with secondary forniceal damage (79, 80), facilitated by antithrombotic therapy (81). Less frequently inflammatory (30, 82), traumatic (83), or toxic (84) etiologies were described (Table 2).

**Table 2:** Clinical characteristics and etiology of reported cases.

Clinical characteristics		
Sex, female	27 (49)	
Age, mean (SD)	58 (14)	
Memory deficit	Anterograde: 40 (73)	Anterograde + retrograde: 15 (27)
Other symptoms	7 (13)	
Headache	9 (16)	
Others cognitive deficits	19 (35)	
Confabulation	7 (13)	
Confusion	13 (24)	

Lesion site	Bilateral	Left	Right	None
Fornix involvement	48 (87)	6 (11)	1 (2)	0 (0)
Corpus callosum	20 (36)	2 (4)	1 (2)	32 (58)
Caudate nuclei	3 (6)	2 (4)	0 (0)	50 (91)
Frontal lobe	1 (2)	1 (2)	2 (4)	51 (93)
Thalamus	0 (0)	1 (2)	1 (2)	53 (96)

Follow-up*			
Follow up, months, median (IQR)	3 (3.5)		
Follow-up outcome	No improvement: 14 (34)	Partial improvement: 17 (42)	Complete recovery: 10 (25)

Etiology	
1**Cerebrovascular diseases**	43 (78)
−Ischemic stroke	40 (73)
− ICH	1 (2)
− AVM hemorrhage	1 (2)
− IVH	1 (2)
2**Neoplasms**	8 (15)
− B cell lymphoma	2 (4)
− III ventricle colloid cyst (acute bleeding after DAPT)	1 (2)
− III ventricle colloid cyst (acute bleeding)	1 (2)
− Neurocytoma III ventricle (acute bleeding).	1 (2)
− Cavernous angioma	1 (2)
− Pineal germinoma	1 (2)
− Spongioblastoma	1 (2)
3**Inflammatory**	2 (4)
− Multiple sclerosis	1 (2)
− Necrotizing encephalitis	1 (2)
4**Traumatic**	1 (2)
− Penetrating injury (gunshot)	1 (2)
5**Toxic**	1 (2)
− Intrathecal methotrexate toxicity	1 (2)

*Follow-up data was available in 41 patients. The data is expressed as count (percentage) unless otherwise specified.

Neuroimaging revealed bilateral forniceal involvement in most patients (48/55, 87%). Corpus callosum (23/55, 42%) and more rarely caudate nuclei (5/55, 9%) and thalamus (2/55, 4%) were also injured.

The amnestic syndrome was characterized by an anterograde amnesia in all patients, which was associated with retrograde amnesia in 15/55 (27%) of cases. The retrograde memory impairment was often limited to the recent past and had a significant temporal gradient, coherently with what previously discussed on the role of the fornix in memory function (85, 86).

Amnesia was often an isolated presentation. Other cognitive deficits were present in 37% of cases, predominantly an executive dysfunction.

Other neurological deficits were quite rare and affected only 14% of patients. Confusion and confabulation were reported, respectively, in 14 (25%) and 8 (14%) of patients. Confabulation was more frequent (60%) in patients with caudate nucleus involvement than in those without caudate nucleus lesions (8%). Confusion was also associated with corpus callosum (CC) involvement and was present in 43% of patients with CC lesions versus 9% of patients without CC damage.

Follow-up data were reported in 41/55 (75%) of patients. No standardized outcome reporting prevented a detailed analysis of the recovery trajectory of memory deficit after the acute phase. However, from available information, the outcome at last follow-up (median follow-up of 3 months) was: no improvement in 14/41 (34%), partial improvement in 17/41 (41%) and complete recovery in 10/41 (23%) patients. The characteristics of reported cases are summarized in Table 2.

### Acute ischemic stroke of the fornix

Considering that ischemic stroke involving the fornix accounted for about ¾ of the cases of acute amnestic syndrome due to fornix lesions, it deserves a deeper analysis. Clinical characteristics of ischemic stroke patients are summarized in Table 3.

**Table 3:** Characteristics of acute ischemic stroke patients.

Clinical characteristics				
Sex, female, *N* (%)	23 (58)			
Age, mean (SD)	59 (14)			
Memory deficit	Anterograde: 27 (68)	Anterograde + retrograde: 13 (33)		
Other symptoms, *N* (%)	3 (8)			
Headache, *N* (%)	5 (13)			
Others cognitive deficits, *N* (%)	15 (38)			
Confabulation, *N* (%)	5 (13)			
Confusion, *N* (%)	11 (27)			
Fornix involvement	Bilateral: 26 (90)	Left: 3 (8)	Right: 1 (3)	
**Vascular territory**				
Subcallosal artery, *N* (%)	38 (95)			
Recurrent artery of heubner, *N* (%)	Bilateral 3 (8)	Left: 1 (3)	Right: 0 (0)	No: 36 (90)
Vascular risk factors*				
Hypertension, *N* (%)	21 (58)			
Dyslipidemia, *N* (%)	9 (25)			
Diabetes, *N* (%)	12 (33)			
Atrial fibrillation, *N* (%)	4 (11)			
Smoke exposure, *N* (%)	9 (25)			
Coronary artery disease, *N* (%)	8 (22)			
Previous stroke, *N* (%)	4 (11)			
Substance abuse, *N* (%)	1 (3)			
Etiology (TOAST)*				
Large artery atherosclerosis, *N* (%)	1 (3)			
Cardioembolic, *N* (%)	3 (9)			
Small vessel disease, *N* (%)	16 (47)			
Other etiologies, *N* (%)	4 (12)			
Cryptogenic, *N* (%)	10 (29)			
Follow-up*				
Follow up, months, median (IQR)	3 (3)			
Follow-up outcome, *N* (%)	No improvement: 7 (26)			
	Partial improvement: 13 (48)			
	Complete recovery: 7 (26)			

*Stroke etiology was reported in 34 cases, vascular risk factors in 36 cases and follow-up data were available in 23 patients.

The term “amnestic syndrome of the subcallosal artery” (ScA) was first proposed by Moussouttas et al. (29) to describe this clinical presentation of ischemic stroke, as ScA is often the culprit vessel.

We retrieved a total of 40 patients with acute ischemic stroke involving the fornix with acute anterograde amnesia that was not better explained by other lesions. Mean (±SD) age of stroke patients was 59 (±14) years and 23 (58%) were females.

**Figure 4:**
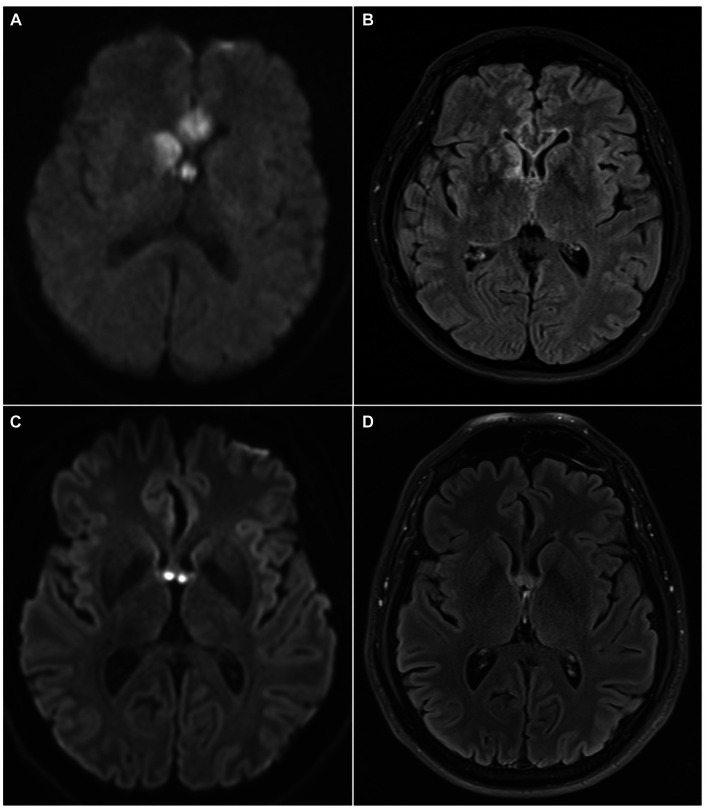
MRI findings of two patients with acute ischemic stroke of the fornix. In the first patient DWI sequence revealed restricted diffusion involving the columns of the fornix, the corpus callosum and the right caudate nucleus **(A)**; these structures are included in the vascular territory of the ScA (fornix, corpus callosum) and the right RAH (caudate head). FLAIR sequence obtained at 1-month shows gliotic lesions evolution **(B)**. In the second patient, MRI acquired at symptom onset reveals acute ischemic stroke involving only forniceal columns both in DWI **(C)** and FLAIR **(D)** sequences. Forniceal columns appear swollen as well. DWI, diffusion weighted imaging; ScA, subcallosal artery; RAH, recurrent artery of Heubner; FLAIR, fluid attenuated inversion recovery.

MRI data were available in all patients and revealed an isolated fornix involvement in 22 (52%) cases, 90% of which were bilateral. The most frequently associated involved structure was the corpus callosum (18, 45%), as expected by the vascular territory of the ScA previously described. Along with ScA involvement, 4/40 (10%) of patients had a concomitant stroke in the territory of recurrent artery of Heuber (RAH). Examples of MRI findings in two cases of acute ischemic stroke of the fornix are reported in Figure 4.

Two cases of vertebrobasilar stroke with fornix involvement have been reported. One case of vertebral artery dissection with subsequent fornix ischemia was attributed to embolism involving the left lateral posterior choroidal artery (38). The presence of vertigo and right lateral homonymous hemianopsia (due to the concomitant embolic occlusion of left P2-PCA) helped in the diagnosis and localization (38). Another case, with right fornix and anteromedial right thalamus infarction was attributed to the occlusion of the tubero-thalamic artery due to its peculiar lesions distribution (56). The memory disturbance was indistinguishable from the other reported cases (38, 56).

Vascular risk factors were reported in 36/40 (90%) of case reports; patients’ medical history was not adequately reported in the remaining 4 cases. As expected, at least one vascular risk factor was reported in the majority of them (28/36, 78%), of which the most prevalent risk factor was arterial hypertension, affecting 21/36 (58%) of patients, followed by diabetes mellitus 12/36 (33%).

Regarding clinical characteristics at presentation, the memory deficit was only anterograde in 27/40 (68%) of patients, while 32% had also retrograde amnesia.

The presence of associated neurological symptoms is an important feature because it can be an essential clue to suspect an ischemic etiology. However, only 3 (8%) of patients had other neurological deficits and 5 (13%) reported headache. A total of 15 (38%) patient had other cognitive deficits, often executive dysfunction, that were usually revealed by subsequent formal neuropsychological testing.

Confusion 11/40 (28%) and confabulation 5/40 (13%) were not infrequently associated with amnesia.

As for the general study population, confabulation was more frequent in cases with RAH involvement which determines caudate nuclei lesion (6% vs. 75%), while confusion in case of reported corpus callosum ischemia (9% vs. 50%).

Stroke etiology was reported in 34/40 (85%) of cases. The predominant etiology according to TOAST classification (32) was small vessel disease (*n* = 16, 47%), followed by other known etiology (*n* = 4, 12%), cardioembolic (*n* = 3, 9%) and large artery atherosclerosis (*n* = 1, 3%); 10 (29%) were cryptogenic. The etiology reported for the 4 patients with other known etiologies were: vasospasm after subarachnoid hemorrhage, giant cell arteritis, vertebral artery dissection and one patient was diagnosed a pulmonary neoplasm during the diagnostic workup for cryptogenic stroke.

Regarding reperfusion therapies, none of the patients was treated with either intravenous thrombolysis or mechanical thrombectomy in the acute phase.

Outcome data were of limited quality and reported in 27/40 (68%) of cases; modified Rankin Scale was not reported in any case. From available information, 7 (25.9%) did not recover, 13 (48.1%) partially recovered, and 7 (25.9%) had complete resolution of symptoms.

## Discussion

Acute amnestic syndrome with anterograde memory deficit is an uncommon clinical presentation and poses a significant diagnostic challenge. To our knowledge, we are reporting the largest and most comprehensive systematic review of case reports of acute amnestic syndrome due to non-iatrogenic lesions of the fornix.

Our review highlights that, apart from surgical cases where the diagnosis is straightforward, several different etiologies should be considered. However, when the symptom onset is acute, as for other neurological syndromes, the most relevant etiology is cerebrovascular disease: this rare stroke syndrome should be considered in these cases. In particular, our study confirm that the ScA is the main culprit vessel, and, in the majority of cases, the etiology of stroke is small vessel disease.

A first significant finding is that the clinical characteristics of the amnestic syndrome associated with fornix lesions seem to be non-specific, and often memory deficit occurs in isolation. As a consequence, the clinical picture widely overlaps with that of more frequent etiologies like Wernicke-Korsakoff syndrome and, especially, TGA. While additional neurological deficits are a strong element against a diagnosis of TGA, they are unfortunately quite rare, especially if fornix alone is involved. These results are in line with previous findings on ischemic amnesia (87).

**Figure 5:**
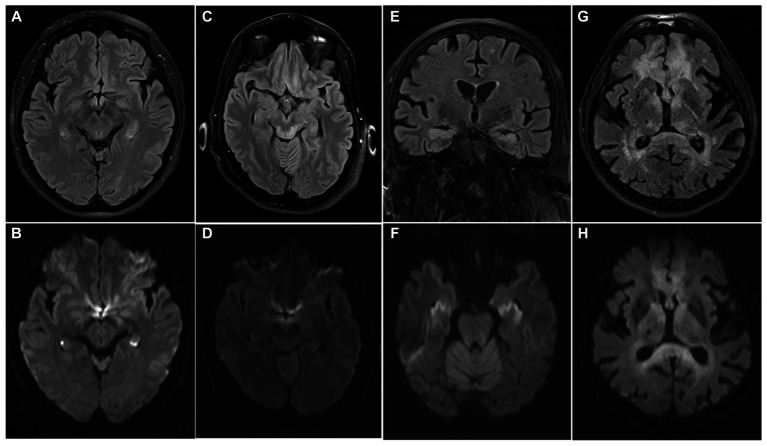
MRI differential diagnosis of acute amnestic syndrome. Transient global amnesia **(A,B)**, Wernicke-Korsakoff syndrome **(C,D)**, limbic encephalitis **(E,F)**, and lymphoma **(G,H)** can present with acute anterograde amnesia, with or without other neurological symptoms. Bilateral HPDLs in a case of TGA are showed **(A)** (FLAIR) and **(B)** (DWI). Typical tectal plate lesions associated with alcoholic Wernicke-Korsakoff syndrome are showed in FLAIR and DWI sequences **(C,D)**. MRI shows bilateral hyperintensity of the hippocampi on both FLAIR **(E)** and DWI **(F)** sequences in a case of paraneoplastic limbic encephalitis. A central nervous system lymphoma involving the fornix, but also frontal lobes and periventricular withe matter is showed in **(G)** (FLAIR) and **(H)** (DWI), appearing as a diffuse hyperintense alteration on both sequences.

Several neurological diseases should be considered in the differential diagnosis. TGA is the most common and typically presents with isolated anterograde amnesia with a limited retrograde component. Focal neurological deficits are absent, and the transient nature of the memory deficit is a key element for diagnosis, but of limited utility during the acute evaluation. MRI can demonstrate hippocampal punctate diffusion lesions (HPDL) (Figures 5A,B) or rarely extra-hippocampal punctate diffusion lesions (E-HPDL), but it can also be normal (88, 89).

Wernicke-Korsakoff syndrome is another mimic, but the medical history often reveals the cause of the thiamine deficit aiding in the diagnostic process; MRI can show hyperintensity in T2/FLAIR in mammillary bodies, medial thalami, hypothalamus, tectal plate, periaqueductal area; rarer in cerebellum, brainstem, basal ganglia (Figures 5C,D) (90, 91).

Transient epileptic amnesia (TEA) is a form of late-onset temporal lobe epilepsy that can present with the same syndrome (92). However, memory deficit is prevalently retrograde and of short duration (<1 h), episodes are often recurrent, and electroencephalography can show epileptiform abnormalities in the hippocampal-mesial temporal lobe regions (93).

Limbic encephalitis (Figures 5E,F), dissociative amnesia and neoplasms as lymphoma (Figures 5G,H) are other potential differential diagnosis but are usually more easily distinguishable from the clinical characteristics, and amnesia is rarely the only neurological symptom. The principal characteristics of the main pathologies included in the differential diagnosis are summarized in Table 4.

**Table 4:** Differential diagnosis of stroke of the fornix.

	Transient global amnesia	Wernicke-Korsakoff syndrome	Transient epileptic amnesia	Limbic encephalitis	Dissociative amnesia
**Epidemiology**	Age: 50–70. Incidence: 3–8/100,000/y. No gender differences (94).	Age: 30-70y, peak at 50-55y. Prevalence 1–3%. M/*F* = 0.7 in non-alcoholics, 6 in alcoholics cases (95).	Age: 60y. Following 1998 diagnostic criteria, ~250 cases reported. M/*F* = 2 (96).	Age and sex: variable according to etiology. Prevalence: 2/100000 (97).	Age: 20-40y (98). Prevalence: 1.8–7.3%. No gender differences (99).
**Risk** **Factors**	History of migraine (100). Triggers: precedent physical/ psychological stress (90% of cases), exposure to anesthetics or drugs of abuse (101).	Thiamine deficiency for alcohol addiction (90%), bariatric surgery, hyperemesis, anorexia nervosa, IBDs, end-stage cancer, hemodialysis, infections (102).	Mostly idiopathic; in some instances, caused by structural pathology or auto-immune epilepsy (103).	Often paraneoplastic, but the neoplasm can be occult at presentation.	Possible co-existence of disorders of personality, anxiety, depression, substance abuse.
**Clinical Features**	Acute anterograde amnesia; limited retrograde component, lasting ≤24 h. No other neurological deficits. Mean duration of episodes 6 h (104). Possible mild vegetative symptoms (headache, nausea, dizziness) in the acute phase (105).	Triad: encephalopathy with disorientation, attention deficits, indifference to the environment, progressive slurred speech, impaired consciousness + oculomotor dysfunction + gait ataxia.	Retrograde (less commonly anterograde) amnestic episodes <1 h, 70% in the morning (106). Possible oral automatisms, epigastric aura, deja-vu, paroxysmal alteration of awareness, and olfactory hallucinations, emotional lability. Secondary generalization in 4–10% (96)	Disorientation, confusion, confabulation, and anterograde amnesia (with retrograde loss of autobiographical memories) developing in days-weeks. Possible seizures, gait disturbances, dysautonomia irritability, mood lability, hallucinations and altered sleep/wake cycles (107).	Acute retrograde amnesia (rarely anterograde) limited to the episodic-semantic autobiographical domain, usually of a stressful nature, with variable loss of personal identity, perceptions, and personal affects, without cognitive impairment (1).
**Neuroimaging** **Findings**	*CT*: negative. *MRI*: negative or single/multiple, unilateral/bilateral hippocampal 1–5 mm abnormalities in DWI/FLAIR visible between 12-48 h for up to 6 days after resolution (108). Rarer alterations are in subcortical white matter and basal ganglia (109).	*CT*: negative. *MRI*: hyperintensity in T2/FLAIR in mammillary bodies, medial thalami, hypothalamus, tectal plate, periaqueductal area; rarer in cerebellum, brainstem, basal ganglia (91). DWI restriction slight or absent (90). Possible contrast-enhancement (110). Alcoholics usually have bilateral lesions with atrophy of mammillary bodies, hippocampus, corpus callosum and cortex (111).	*CT*: hippocampal calcifications in 1/3 of cases. *MRI*: diffuse hippocampal signal abnormality and swelling in T2/DWI (112). Atrophy in both hippocampi, perirhinal and orbitofrontal cortices (113).	*CT*: negative. *MRI*: bilateral hyperintensities in medial temporal lobes in T2/FLAIR. Sometimes unilateral or no alterations (114). Swelling and hyperintensity may persist over months to years and later lead to atrophy (115). DWI restriction and ill-defined contrast enhancement can be observed.	*CT*: negative. *MRI*: negative.
**EEG**	Normal or paroxysmal abnormalities (described periodic lateralized epileptiform discharges, left temporal sharp-wave and central spike and wave discharges) (73).	Diffuse background slowing with low-voltage theta and delta activity over the fronto-temporal brain regions as the severity of the encephalopathy increases, at times without reactivity to external stimuli (116).	Clear epileptiform abnormalities in 37% of cases with bilateral or preferentially left-side discharges involving the hippocampal-mesial temporal lobe regions (93).	Generalized slow-wave activity, with temporal epileptiform discharges, and uni-or bilateral frontotemporal slow-wave activity (117).	Normal.
**Outcome**	No increased risk of mortality, epilepsy or stroke (118). Some subclinical deficits in episodic and semantic memory may persist also after years (94).Recurrency in 15% of patients (119).	Symptoms may be reverted by thiamine in 20% of patients, but chronic deficiency leads atrophy of affected areas, persistent memory and learning deficits (90). Recurrency in 6% of cases (120).	Benign prognosis: at present, it is still debated if medically refractory seizures determine a higher risk for long-term cognitive impairment (121).	Most patients recover after treatment. Relapses may occur.	Time-course of the dissociative episode is variable from patient to patient. In some cases, spontaneously recedes, while in others may become chronic or worsen over time and can recur (99).

DWI: diffusion weighted imaging; ScA: subcallosal artery; RAH: recurrent artery of Heubner; FLAIR: fluid attenuated inversion recovery.

Our analysis has also shown that extraforniceal involvement (e.g., corpus callosum, caudate nuclei) seems to be associated with higher prevalence of additional clinical features, like confabulation and confusion. In cases of ischemic stroke, the involvement of RAH territory is often responsible of this clinical presentation. Confabulation in the context of caudate nuclei stroke has previously been reported and may explain these findings (122). Moreover, the concomitant ScA and RAH involvement has already been proposed as a variable that can aggravate the clinical picture in patients with fornix infarction following AcoA aneurysms treatment (123) and our study supports this finding. Other focal neurological deficits are rare and point to the involvement of other structures outside the limbic system, often in the context of embolism (38).

Another point that emerges is that, while this syndrome has been previously named “amnestic syndrome of the subcallosal artery” (29), it can also occur as a consequence of a posterior circulation stroke in exceedingly rare cases (38, 56). The presence of neurological deficits typical of posterior circulation involvement as ataxia or visual field deficits point to this uncommon circumstance (38).

A striking finding is that in the reported ischemic stroke cases, reperfusion therapy was never administered in the acute phase. This is probably due to a combination of delayed presentation to ED and delayed diagnosis. This is in line with previous literature on ischemic stroke presenting with amnesia, where Michel et al. reported that an ischemic etiology was considered in only 39% of cases (87). The main differential diagnosis was TGA, and many patients had an indistinguishable neurological presentation. Minor focal neurological signs, higher age, and more vascular risk factors were identified as potential clues to reach the correct diagnosis, but the discrimination based on the clinical syndrome was poor (87). Also in this cohort, ischemic patients had a high prevalence of vascular risk factors, but they were often quite young with a mean age of less than 60 years, further complicating the diagnosis. Considering that in most cases the etiology is small vessel disease, endovenous thrombolysis would be the only valid reperfusion therapy and the therapeutic window is narrow; therefore, waiting for the resolution of symptoms to confirm a TGA diagnosis is not possible.

Our study and previous ones on ischemic amnesia demonstrate that the use of MRI in the acute phase of the disease could be useful to assist clinician and would be especially valuable in this rare stroke presentation, where the decision based on the clinical data could be difficult.

**Figure 6:**
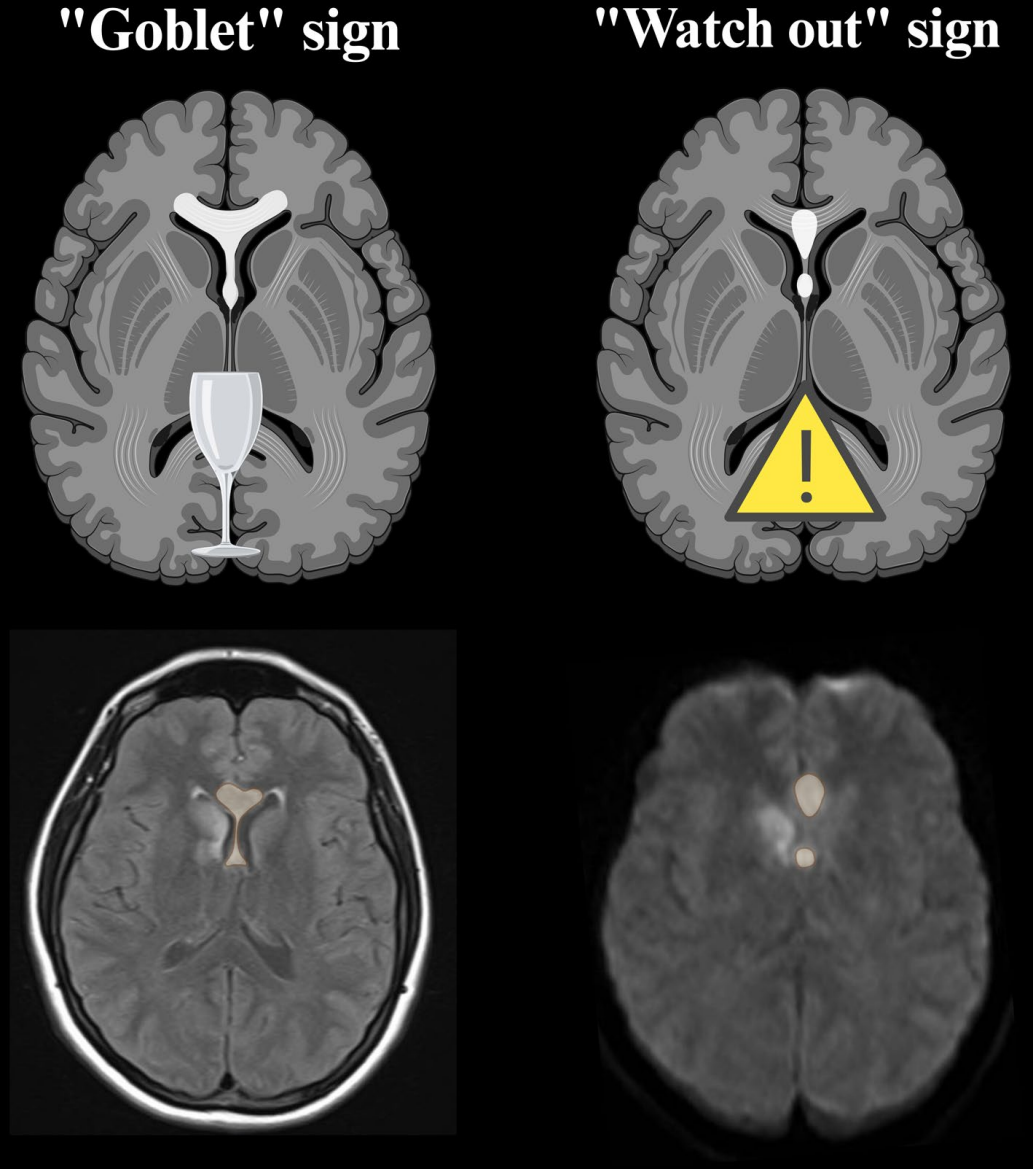
Neuroradiological signs proposed to rapidly recognize the lesional pattern of ScA stroke. They are best visualized on axial DWI sequence in the acute phase (60, 64). The possible concomitant involvement of RAH territory can occur in association with ScA stroke, as can be seen in the MRI images of our cases (created with BioRender.com). DWI, diffusion weighted imaging; ScA, subcallosal artery; RAH, recurrent artery of Heubner.

As the need for a timely diagnosis clearly emerges from the literature, two useful radiological signs should be kept in mind to rapidly identify the peculiar appearance of acute ScA stroke in DWI sequence. The “goblet” sign (64) and the “watch out” sign (60) can be identified on axial images and are due to the fornix columns and the genu of the corpus callosum involvement, that are the main areas involved in ScA stroke (Figure 6). Beside these highly recognizable patterns, the use of 3D MRI sequences has been suggested to detect smaller infarcts (94).

In our study, only 26% of patients had a complete recovery at last follow-up, with three quarter of them having persistent memory deficit. Moreover, a rapid resolution of symptoms was extremely uncommon, as only one patient with a vascular etiology in our cohort had transient symptoms and was a case of hemorrhage and not of fornix infarction (70). The persistence of the amnestic syndrome in our cases confirms the crucial role of the fornix in the Papez circuit. The quality follow-up data is limited, so no definitive conclusions can be drawn.

Overall, the reported cases and especially data pointing to a non-specific clinical presentation, potential worse outcome, and absent reperfusion therapies utilization, strongly suggest the need to maintain a high index of suspicion to rapidly identify and timely treat patients with this rare stroke syndrome. Patients with different etiologies from ischemic stroke can also benefit from treatment and at least a partially restored memory function has been reported (75, 95).

The strengths of our review include a comprehensive systematic literature search, with specific criteria for inclusion and quality appraisal. However, this study has several limitations. Our findings are limited by the quality of reported data; this is especially relevant for the description of unspecific symptoms as confabulation and confusion. Not uniform follow-up and outcome reporting is another significant limitation. Most importantly, case series and reports are uncontrolled, and while they can suggest hypotheses, they cannot establish definitive associations and they are not robust enough for statistical inference.

## Conclusion

Acute amnestic syndrome can be rarely caused by fornix lesions. In most cases of acute/subacute presentation the etiology is vascular, mainly caused by acute ischemic stroke involving the ScA territory. The differential diagnosis is challenging and the distinction from common mimics as TGA is often impossible only on a clinical basis. A high index of suspicion should be maintained to avoid misdiagnosis of time-dependent diseases and to provide adequate acute treatment. A significant percentage of patients with amnestic syndrome due to fornix lesions seems to have persistent memory deficits at follow-up. However, due to the limited quality of available data, more studies are needed to better understand their outcome and identify prognostic factors in patients with amnestic syndrome due to fornix lesions.

## Data availability statement

The original contributions presented in the study are included in the article/Supplementary material, further inquiries can be directed to the corresponding author.

## Author contributions

FM: Conceptualization, Data curation, Formal analysis, Investigation, Methodology, Visualization, Writing – original draft, Writing – review & editing, Funding acquisition. FF: Conceptualization, Data curation, Investigation, Methodology, Visualization, Writing – original draft, Writing – review & editing. AM: Visualization, Writing – review & editing, Conceptualization, Methodology. YM: Writing – review & editing, Data curation. MG: Visualization, Writing – review & editing, Conceptualization, Supervision. ACo: Supervision, Writing – review & editing. AP: Supervision, Validation, Writing – review & editing, Conceptualization, Data curation. ACa: Funding acquisition, Supervision, Validation, Writing – review & editing, Conceptualization.
