# Supplementary material for: Acute amnestic syndrome in fornix lesions: a systematic review of reported cases with a focus on differential diagnosis
Source: Front Neurol. 2024 Jan 25;15:1338291. doi: 10.3389/fneur.2024.1338291 (PMC10850356; doi:10.3389/fneur.2024.1338291)
Supplement: Supplementary file 1 [file Table_1.docx]

Supplementary Material

**Supplementary Table 1.** Research terms.

| **PUBMED** | | |
| --- | --- | --- |
| # 1 | fornix [MeSH Terms] OR fornix OR fornices | 6440 |
| # 2 | amnesia OR amnestic OR memory deficit OR amnesia [MeSH Terms] | 101964 |
| # 3 | stroke OR infarction OR ischemia OR stroke [MeSH Terms] | 941808 |
| # 4 | # 1 AND (#2 OR #3) | 565 |
| **SCOPUS** | | |
|  | TITLE-ABS-KEY (fornix OR fornices) AND (TITLE-ABS-KEY (amnesia OR amnestic OR ( memory AND deficit ) ) OR TITLE-ABS-KEY ( stroke OR infarction OR ischemia ) ) | 1166 |
| **Web of Science** | | |
| # 1 | ALL=(fornix) OR ALL=(fornices) | 699 |
| # 2 | ALL=(amnesia) OR ALL=(amnestic) OR ALL= (memory deficit) | 91,442 |
| # 3 | ALL=(stroke) OR ALL=(infarctions) OR ALL=(ischemia) | 1184787 |
| # 4 | # 1 AND (#2 OR #3) | 1097 |

**Supplementary Table 2.** Studies quality appraisal. NIH quality assessment tool for case series/case reports.

(A) No = no provided information; Partially = some information is provided; Yes = presence of objective rating scales of memory and cognitive deficits.

(B) No = if follow-up was < 3 months without improvement; Yes = if follow-up was >/= 3 months, or < 3 months with complete recovery.

(C) No = no provided information; Partially = information is provided either incomplete or without follow-up data; Yes = presence of clinical and neuroimaging finding, cognitive assessment, follow-up information.

Abbreviations: %: not applicable to case reports; NR: information not reported.

| **Autor** | **year** | **study_type** | **Was the study question or objective clearly stated?** | **Was the study population clearly and fully described, including a case definition?** | **Were the cases consecutive?** | **Were the subjects comparable?** | **Was the intervention clearly described?** | **Were the outcome measures clearly defined, valid, reliable, and implemented consistently across all study participants? (A)** | **Was the length of follow-up adequate? (B)** | **Were the statistical methods well-described?** | **Were the results well-described? (C)** |
| --- | --- | --- | --- | --- | --- | --- | --- | --- | --- | --- | --- |
| Abu-Alya A.I. et al.  (70) | 2023 | Case report | Yes | Yes | % | % | % | Partially | Yes | % | Partially |
| Adamovich B.L. et al.  (42) | 2009 | Case report | No | Yes | % | % | % | No | No | % | Partially |
| Akpınar C.K. and Sayılır I.  (67) | 2017 | Case report | Yes | Yes | % | % | % | Partially | No | % | Partially |
| Boardman J. and Zermansky A.  (60) | 2019 | Case report | No | Yes | % | % | % | Yes | NR | % | Partially |
| Carota A. et al.  (26) | 2013 | Case report | No | Yes | % | % | % | Yes | NR | % | Partially |
| Chen J.Y. et al.  (46) | 2008 | Case report | No | Yes | % | % | % | No | NR | % | Partially |
| Cho M.J. et al.  (57) | 2020 | Case report | Yes | Yes | % | % | % | No | Yes | % | Partially |
| D'Esposito M. et al.  (81) | 1995 | Case report | Yes | Yes | % | % | % | Yes | Yes | % | Yes |
| den Heijer T. et al.  (47) | 2007 | Case report | Yes | Yes | % | % | % | Yes | NR | % | Partially |
| Ghannam M. et al.  (54) | 2021 | Case series | Yes | Yes | NR | Yes | % | Partially | Yes | % | Partially |
| Gupta M. et al.  (37) | 2014 | Case report | Yes | Yes | % | % | % | Yes | Yes | % | Yes |
| Heilman K.M. and Sypert G.W.  (76) | 1977 | Case report | Yes | Yes | % | % | % | Yes | Yes | % | Yes |
| Holla V.V. et al.  (58) | 2020 | Case report | Yes | Yes | % | % | % | Yes | Yes | % | Yes |
| Izgi E. et al.  (50) | 2023 | Case report | Yes | Yes | % | % | % | No | NR | % | Partially |
| Jiang J. et al.  (55) | 2021 | Case report | No | Yes | % | % | % | Yes | NR | % | Partially |
| Kannath S.K. et al.  (66) | 2017 | Case report | Yes | Yes | % | % | % | No | Yes | % | Partially |
| Karri M. et al.  (59) | 2019 | Case report | No | Yes | % | % | % | Partially | Yes | % | Partially |
| Katoh M. et al.  (72) | 2013 | Case report | Yes | Yes | % | % | % | Yes | Yes | % | Yes |
| Korematsu K. et al.  (41) | 2010 | Case report | Yes | Yes | % | % | % | Yes | Yes | % | Yes |
| Kurokawa T. et al.  (36) | 2015 | Case report | Yes | Yes | % | % | % | Yes | No | % | Yes |
| Kwan B.Y.M. et al.  (82) | 2015 | Case report | Yes | Yes | % | % | % | Partially | Yes | % | Yes |
| Kwon H.G. and Jang S.H.  (71) | 2013 | Case report | Yes | Yes | % | % | % | Yes | No | % | Yes |
| Magnin E. et al.  (28) | 2016 | Case report | No | Yes | % | % | % | Yes | Yes | % | Partially |
| Meila D. et al.  (38) | 2015 | Case series | Yes | Yes | NR | Partially | % | Yes | NR | % | Partially |
| Miranda M.O. et al.  (34) | 2015 | Case report | Yes | Yes | % | % | % | Partially | NR | % | Partially |
| Moudgil S.S. et al.  (52) | 2000 | Case report | Yes | Yes | % | % | % | Partially | No | % | Partially |
| Moussouttas M. et al.  (27) | 2005 | Case report | Yes | Yes | % | % | % | Yes | Yes | % | Yes |
| Murr N. et al.  (40) | 2012 | Case report | Yes | Yes | % | % | % | No | NR | % | Partially |
| Nishibayashi H. et al.  (78) | 2006 | Case report | Yes | Yes | % | % | % | Yes | Yes | % | Yes |
| Pardina-Vilella L. et al. (62) | 2018 | Case report | No | Yes | % | % | % | Partially | Yes | % | Partially |
| Park S.A. et al.  (53) | 2000 | Case report | Yes | Yes | % | % | % | Yes | Yes | % | Yes |
| Qi-Huang S. et al.  (49) | 2022 | Case report | Yes | Yes | % | % | % | Yes | NR | % | Partially |
| Ren C. et al.  (45) | 2018 | Case report | Yes | Yes | % | % | % | Partially | Yes | % | Partially |
| Renou P. et al.  (43) | 2008 | Case report | No | Yes | % | % | % | No | Yes | % | Partially |
| Rizek P. et al.  (39) | 2013 | Case report | Yes | Yes | % | % | % | Yes | Yes | % | Yes |
| Saito Y et al.  (48) | 2006 | Case report | Yes | Yes | % | % | % | Yes | NR | % | Partially |
| Salvalaggio A. et al.  (64) | 2018 | Case series | Yes | Yes | NR | Yes | % | Partially | NR | % | Partially |
| Sidra M. et al.  (56) | 2021 | Case report | No | Yes | % | % | % | No | No | % | No |
| Sivakumaran R. and Edwards R.J.  (77) | 2015 | Case report | No | Yes | % | % | % | No | Yes | % | Partially |
| Snyder R. et al.  (79) | 2022 | Case report | Yes | Yes | % | % | % | Partially | Yes | % | Yes |
| Takahashi M. et al.  (69) | 2016 | Case report | No | Partially | % | % | % | Yes | Yes | % | Partially |
| Takano Y. et al.  (61) | 2018 | Case report | Yes | Yes | % | % | % | Yes | No | % | Yes |
| Thapa K. et al.  (33) | 2023 | Case report | No | Yes | % | % | % | Partially | NR | % | Partially |
| Turine G. et al.  (35) | 2016 | Case report | No | Yes | % | % | % | Partially | Yes | % | Yes |
| Verfaellie et al.  (73) | 1991 | Case report | Yes | Yes | % | % | % | Yes | Yes | Yes | Yes |
| Wang J. et al.  (44) | 2018 | Case report | Yes | Yes | % | % | % | Yes | Yes | % | Yes |
| Yamamoto T. et al.  (80) | 1990 | Case report | Yes | Yes | % | % | % | Partially | Yes | % | Yes |
| Ji Y. et al.  (68) | 2020 | Case series | Yes | Yes | NR | No | % | Yes | Yes | % | Yes |
| Yoon S.-S. et al.  (74) | 2008 | Case report | Yes | Yes | % | % | % | Yes | Yes | % | Yes |
| Yoshida M. et al.  (75) | 2016 | Case report | No | Partially | % | % | % | Yes | Yes | % | Yes |
| Zhang H. et al.  (51) | 2022 | Case report | Yes | Yes | % | % | % | Yes | Yes | % | Yes |
| Zhu Q.Y. et al.  (63) | 2018 | Case report | No | Partially | % | % | % | Yes | NR | % | No |

**Supplementary Table 3**. Included cases of amnesia due to fornix lesions.

Abbreviations: a, anterograde amnesia; r, retrograde amnesia; AF, atrial fibrillation; CAD, coronary artery disease; DM, diabetes mellitus; HP, hypercholesterolemia; HTN, hypertension; NA, information not reported.

| **Author** | **Sex,**  **age** | **Amnesia** | **Other symptoms** | **Headache** | **Other cognitive symptoms** | **Confabulation** | **Confusion** | **Etiology** | **Lesion location** | **ScA** | **RAH** | **Other Involved Vessels** | **CVD risk factors** | **Stroke etiology** | **Follow-up (Months)** | **Recovery** |
| --- | --- | --- | --- | --- | --- | --- | --- | --- | --- | --- | --- | --- | --- | --- | --- | --- |
| **Abu-Alya A.I. et al.**(70) | m, 65 | a | no | no | no | no | no | ICH | Bilateral fornix. | NA | NA | NA | NA | NA | 4 | complete |
| **Adamovich B.L. et al.** (42) | f, 53 | a+r | no | no | no | no | no | ischemic stroke | Bilateral fornix. | bilateral | not involved | none | NA | Small vessel disease | 2 | none |
| **Akpınar C.K. and Sayılır I.** (67) | f, 75 | a | no | no | no | no | no | ischemic stroke | Bilateral fornix. | bilateral | not involved | none | HTN, DM | NA | 1 | partial |
| **Boardman J. and Zermansky A.** (60) | m, 80 | a | no | no | Hallucinations at night | yes | yes | ischemic stroke | Bilateral fornix. | bilateral | not involved | none | HTN, DM | Small vessel disease | NA | NA |
| **Carota A. et al.** (26) | m, 80 | a | no | no | no | yes | no | B cell lymphoma | Bilateral fornix. | NA | NA | NA | NA | NA | NA | NA |
| **Chen J.Y. et al.** (46) | f, 72 | a | Mild right facial droop, right arm weakness, dyssynergia and numbness | no | Psychomotor retardation | no | no | ischemic stroke | Bilateral fornix. | bilateral | not involved | none | HTN, HP, DM, CAD | Small vessel disease | NA | NA |
| **Cho M.J. et al.** (57) | f, 32 | a | no | yes | no | no | no | ischemic stroke | Bilateral fornix. | bilateral | not involved | none | no | Undetermined | 1 | complete |
| **D'Esposito M. et al.** (81) | f, 32 | a | Right hemiparesis.  Severe proprioceptive loss on her left side and left homonymous hemianopsia | no | Conduction aphasia. Executive dysfunction. | no | no | Penetrating injury (gunshot) | Bilateral fornix; posterior parietal lobe. | NA | NA | NA | NA | NA | 12 | none |
| **den Heijer T. et al.** (47) | f, 76 | a | no | no | Mild dyspraxia of the left hand | yes | no | ischemic stroke | Bilateral fornix, corpus callosum and caudate nuclei. | bilateral | bilateral | none | NA | NA | NA | NA |
| **Ghannam M. et al.** (54) | f, 71 | a+r | no | no | no | no | yes | ischemic stroke | Bilateral fornix, corpus callosum and caudate nuclei; right frontal lobe | bilateral | bilateral | right ACA | HTN, HP, CAD | Undetermined | 3 | partial |
| **Ghannam M. et al.** (54) | m, 56 | a | no | no | no | no | no | ischemic stroke | Right fornix and thalamus. | not involved | not involved | Tubero-thalamic artery | HTN, HP, DM, Smoke, previous stroke | Undetermined | NA | NA |
| **Ghannam M. et al.**  (54) | m, 61 | a+r | no | no | Ideomotor apraxia and right-left confusion. | yes | yes | ischemic stroke | Bilateral fornix, corpus callosum and left caudate nucleus; right frontal lobe. | bilateral | left | aca r | HTN, HP, DM, Smoke | Cardioembolic | 10 | none |
| **Gupta M. et al.**  (37) | f, 60 | a+r | no | yes | no | no | yes | ischemic stroke | Left fornix. | left | not involved | none | HP, AF, CAD, Previous stroke | Small vessel disease | 1 | complete |
| **Heilman K.M. and Sypert G.W.**  (76) | f, 42 | a | no | yes | no | no | no | Spongioblastoma | Bilateral fornix. | NA | NA | NA | NA | NA | 5 | none |
| **Holla V.V. et al.**  (58) | f, 55 | a | no | no | no | no | yes | ischemic stroke | Bilateral fornix and corpus callosum. | bilateral | not involved | none | HTN | Small vessel disease | 3 | partial |
| **Izgi E. et al.**  (50) | f, 20 | a | paraparesis/paresthesia feet | no | no | no | no | ischemic stroke | Bilateral fornix | bilateral | not involved | none | no | Undetermined | NA | NA |
| **Jiang J. et al.**  (55) | f, 49 | a | no | no | Executive dysfunction | no | no | ischemic stroke | Bilateral fornix | bilateral | not involved | none | no | Small vessel disease | NA | NA |
| **Kannath S.K. et al.**  (66) | m, 60 | a | no | no | Impaired consciousness. | no | yes | ischemic stroke | Bilateral fornix | bilateral | not involved | none | no | Other cause (Vasospasm after SAH) | 3 | complete |
| **Karri M. et al.**  (59) | m, 50 | a | no | no | no | no | no | ischemic stroke | Bilateral fornix | bilateral | not involved | none | no | Undetermined | 3 | complete |
| **Katoh M. et al.**  (72) | m, 44 | a | no | no | no | no | no | cavernous angioma | Bilateral fornix | NA | NA | NA | NA | NA | 5 | partial |
| **Korematsu K. et al.**  (41) | m, 52 | a+r | no | no | no | no | no | ischemic stroke | Left fornix. | left | not involved | none | Smoke | NA | 3 | partial |
| **Kurokawa T. et al.** (36) | f, 26 | a+r | vertigo; right homonymous hemianopia, sensory ataxia | yes | no | no | no | ischemic stroke | Bilateral fornix and left caudate nucleus . Occipital lobe | not involved | not involved | Lateral posterior choroidal artery/medial posterior choroidal artery/tubero-thalamic artery, left posterior cerebral artery. | no | Other cause (dissection) | 1 | partial |
| **Kwan B.Y.M. et al.** (82) | m, UNK | a | no | no | no | no | no | drug toxicity (intrathecal methotrexate) | Bilateral fornix and corpus callosum. | NA | NA | NA | NA | NA | 2 | none |
| **Kwon H.G. and Jang S.H.**  (71) | m, 63 | a | no | no | no | no | no | Intraventricular hemorrhage | Bilateral fornix | bilateral | not involved | none | NA | NA | 2,5 | none |
| **Magnin E. et al.** (28) | m, 37 | a | no | no | no | no | no | Multiple sclerosis | Left fornix and corpus callosum. Brain and cervical demyelinating lesions. | NA | NA | NA | NA | NA | 12 | none |
| **Meila D. et al.** (38) | m, 62 | a+r | no | no | no | no | no | ischemic stroke | Bilateral fornix and corpus callosum. | bilateral | not involved | none | NA | Small vessel disease | NA | NA |
| **Miranda M.O. et al.**  (34) | f, 56 | a+r | no | no | Disinhibition. Slightly dysphasic. | yes | no | ischemic stroke | Bilateral fornix, corpus callosum and caudate nuclei. | bilateral | bilateral | none | Smoke | Undetermined | NA | NA |
| **Moudgil S.S. et al.**  (52) | f, 71 | a | no | no | no | no | no | ischemic stroke | Bilateral fornix and corpus callosum and frontal lobes. | bilateral | not involved | NA | HTN | Small vessel disease | 1 | partial |
| **Moussouttas M. et al.** (27) | m, 61 | a | no | no | Executive dysfunction | no | yes | ischemic stroke | Bilateral fornix and corpus callosum; left frontal lobe. | bilateral | not involved | Left anterior cerebral artery. | HTN, HP, CAD | Cardioembolic | 5 | none |
| **Murr N. et al.** (40) | f, 60 | a | no | yes | no | no | yes | ischemic stroke | Bilateral fornix and corpus callosum. | bilateral | not involved | none | HTN, Smoke | Other cause (giant cell arteritis) | NA | NA |
| **Nishibayashi H. et al.** (78) | m, 43 | a | no | no | Executive dysfunction | no | no | Neurocytoma III ventricle with acute bleeding. | Left fornix. | NA | NA | NA | NA | NA | 10 | complete |
| **Pardina-Vilella L. et al.** (62) | m, 79 | a | no | no | no | no | no | ischemic stroke | Bilateral fornix and corpus callosum. | bilateral | not involved | none | no | Cardioembolic | 3 | none |
| **Park S.A. et al.** (53) | f, 60 | a | no | no | no | no | no | ischemic stroke | Bilateral fornix and corpus callosum (right) | bilateral | not involved | none | HTN, DM | Small vessel disease | 4 | partial |
| **Qi-Huang S. et al.** (49) | m, 60 | a | no | no | no | no | yes | ischemic stroke | Left fornix. | bilateral | not involved | none | HTN, previous stroke, substance abuse | Small vessel disease | NA | NA |
| **Ren C. et al.** (45) | f, 66 | a | no | no | 1 | no | no | ischemic stroke | Bilateral fornix | bilateral | not involved | none | HTN, HP | Athero-thrombotic | 0,3 | complete |
| **Renou P. et al.**  (43) | m, 68 | a+r | no | no | no | no | no | ischemic stroke | Bilateral fornix | bilateral | not involved | none | HTN, DM, CAD | NA | 12 | none |
| **Rizek P. et al.**  (39) | m, 56 | a | no | no | Visuospatial and executive dysfunction. | no | no | ischemic stroke | Bilateral fornix | bilateral | not involved | none | AF, smoke, previous stroke | Small vessel disease | 4 | none |
| **Saito Y. et al.**  (48) | f, 71 | a | no | no | Apathy | no | no | ischemic stroke | Bilateral fornix and corpus callosum. | bilateral | not involved | none | HTN, smoke | Small vessel disease | NA | NA |
| **Salvalaggio A. et al.**  (64) | m, 59 | a | no | no | Disinhibition | no | no | ischemic stroke | Bilateral fornix | bilateral | not involved | none | HTN | Undetermined | NA | partial |
| **Salvalaggio A. et al.**  (64) | m, 61 | a | no | no | Executive dysfunction | yes | no | ischemic stroke | Bilateral fornix | bilateral | not involved | none | HTN, HP, Smoke, CAD | Other cause (paraneoplastic) | NA | partial |
| **Sidra M. et al.** (56) | m, 52 | a | no | no | no | no | no | ischemic stroke | Bilateral fornix | bilateral | not involved | none | no | Small vessel disease | 1 | partial |
| **Sivakumaran R. and Edwards R.J.** (77) | m, 48 | a | no | yes | no | no | no | Colloid cyst hemorrhage | Bilateral fornix | NA | NA | NA | NA | NA | 2 | complete |
| **Snyder R. et al.** (79) | m, 65 | a | Gait ataxia, urinary incontinence | no | no | no | yes | III ventricle colloid cyst bleeding after double antiplatelet therapy. | Bilateral fornix | NA | NA | NA | NA | NA | 6 | partial |
| **Takahashi M. et al.** (69) | f, 51 | a+r | no | no | no | no | no | Digital subtraction angiography | Bilateral fornix | not involved | involved | none | no | N/R | 8 | complete |
| **Takano Y. et al.** (61) | m, 54 | a+r | no | no | no | no | no | ischemic stroke | Bilateral fornix | bilateral | not involved | none | HTN, DM | Small vessel disease | 1 | partial |
| **Thapa K. et al.** (33) | m, 37 | a | no | no | no | no | no | ischemic stroke | Bilateral fornix | bilateral | not involved | none | no | Undetermined | NA | NA |
| **Turine G. et al.** (35) | f, 74 | a | no | yes | no | no | no | ischemic stroke | Bilateral fornix and corpus callosum. | bilateral | not involved | none | HTN, HP | Undetermined | 3 | none |
| **Verfaellie M. et al.** (73) | m, 39 | a+r | Headache | yes | no | no | no | Arteriovenous malformation hemorrhage | Left fornix and corpus callosum | NA | NA | NA | NA | NA | 7 | partial |
| **Wang J. et al.** (44) | f, 51 | a+r | no | no | no | no | no | ischemic stroke | Bilateral fornix | bilateral | not involved | none | DM | Small vessel disease | 3 | partial |
| **Yamamoto T. et al.** (80) | f, 82 | a | no | yes | Behavioral disturbance | no | yes | Necrotizing encephalitis | Bilateral fornix and corpus callosum. Bilateral hippocampus | NA | NA | NA | NA | NA | 4 | none |
| **Ji Y. et al.** (68) | f, 68 | a | no | no | Dyscalculia | no | no | ischemic stroke | Bilateral fornix and corpus callosum. | not involved | bilateral | none | HP, AF, previous stroke | N/R | 1 | partial |
| **Yoon S.-S. et al.** (74) | m, 65 | a+r | no | no | Executive dysfunction. Apathy. | yes | no | B cell lymphoma | Bilateral fornix and corpus callosum. Hippocampal commissure | NA | NA | NA | NA | NA | 3 | none |
| **Yoshida M. et al.** (75) | f, 45 | a | Diplopia | no | no | no | no | Pineal germinoma | Bilateral fornix | NA | NA | NA | NA | NA | 12 | partial |
| **Zhang H. et al.** (51) | f, 66 | a | no | no | Comprehension and communication deficits; reduce global cognitive performance (MMSE 9/30) | no | yes | ischemic stroke | Bilateral fornix and corpus callosum. | bilateral | not involved | none | HTN, DM | Small vessel disease | 12 | complete |
| **Zhu Q.Y. et al.** (63) | f, 78 | a+r | no | no | no | no | yes | ischemic stroke | Bilateral fornix and corpus callosum. | bilateral | not involved | none | NA | NA | NA | NA |
